# Novel insights into the interaction between N6‐methyladenosine methylation and noncoding RNAs in musculoskeletal disorders

**DOI:** 10.1111/cpr.13294

**Published:** 2022-06-23

**Authors:** Juanjuan Han, Hui Kong, Xueqiang Wang, Xin‐an Zhang

**Affiliations:** ^1^ College of Kinesiology Shenyang Sport University Shenyang China; ^2^ Department of Sport Rehabilitation Shanghai University of Sport Shanghai China; ^3^ Department of Rehabilitation Medicine Shanghai Shangti Orthopaedic Hospital Shanghai China

## Abstract

**Background:**

Musculoskeletal disorder (MSD) are a class of inflammatory and degener‐ative diseases, but the precise molecular mechanisms are still poorly understood. Noncoding RNA (ncRNA) N6‐methyladenosine (m6A) modification plays an essential role in the pathophysiological process of MSD. This review summarized the interaction between m6A RNA methylation and ncRNAs in the molecular regulatory mechanism of MSD. It provides a new perspective for the pathophysiological mechanism and ncRNA m6A targeted therapy of MSD.

**Methods:**

A comprehensive search of databases was conducted with musculoskeletal disorders, noncoding RNA, N6‐methyladenosine, intervertebral disc degeneration, osteoporosis, osteosarcoma, osteoarthritis, skeletal muscle, bone, and cartilage as the key‐words. Then, summarized all the relevant articles.

**Results:**

Intervertebral disc degeneration (IDD), osteoporosis (OP), osteosarcoma (OS), and osteoarthritis (OA) are common MSDs that affect muscle, bone, cartilage, and joint, leading to limited movement, pain, and disability. However, the precise pathogenesis remains unclear, and no effective treatment and drug is available at present. Numerous studies confirmed that the mutual regulation between m6A and ncRNAs (i.e., microRNAs, long ncRNAs, and circular RNAs) was found in MSD, m6A modification can regulate ncRNAs, and ncRNAs can also target m6A regulators. ncRNA m6A modification plays an essential role in the pathophysiological process of MSDs by regulating the homeostasis of skeletal muscle, bone, and cartilage.

**Conclusion:**

m6A interacts with ncRNAs to regulate multiple biological processes and plays important roles in IDD, OP, OS, and OA. These studies provide new insights into the pathophysiological mechanism of MSD and targeting m6A‐modified ncRNAs may be a promising therapy approach.

## Graphical abstract

m6A regulates the expression and function of ncRNAs (i.e., miRNAs, lncRNAs and circRNAs), ncRNAs can also affect m6A‐related protein. m6A‐related proteins in ncRNA are abnormally expressed and closely associated with bone osteogenic, osteoclastogenic processes and myogenesis, participating in modulating the homoeostasis of skeletal muscle, bone and cartilage. ncRNA m6A modification regulates the pathological and physiological processes of musculoskeletal disorders (i.e., IDD, OP, OS and OA). In IDD, ncRNA m6A modification regulated NP cells glucose metabolism, senescence and pyroptosis. In OP, ncRNA m6A modification regulated BMSCs osteogenic differentiation, osteoblast proliferation, differentiation and bone formation. In OS, ncRNA m6A modification regulated OS cells growth, migration, proliferation and apoptosis. In OA, ncRNA m6A modification regulated chondrocyte apoptosis and ECM degradation.

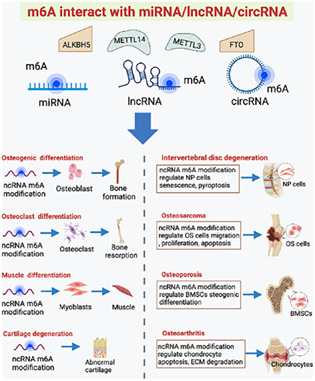

## 1 INTRODUCTION

N6‐methyladenosine (m6A) is the most common internal modification of messenger RNAs (mRNAs)^1^, ^2^ and is widely present in yeast,^3^, ^4^ plants,^5^, ^6^ flies,^7^, ^8^ mammals^9^, ^10^ and viral RNAs.^11^ As the most abundant chemical modification in mammals, m6A modifies about one‐third of mammalian mRNAs, and 3–5 m6A modification sites are present per mRNA on average.^12^ Studies confirmed that m6A modification sites are predominantly distributed in stop codons, 3′untranslated regions (3′UTRs),^13^, ^14^ precursor mRNA, coding sequence and inner long exon of matured mRNA.^15^ In recent years, the understanding of m6A modification in RNA has been further improved with the application of next‐generation sequencing and high‐throughput identification.^13^, ^15^ m6A RNA modification is also widely found in noncoding RNAs (ncRNAs). NcRNA is a class of RNAs that have no protein‐coding ability but is involved in complex gene expression processes^16^ and associated with the pathophysiological process of many diseases.^17^ Evidence found that m6A can regulate the expression and function of ncRNAs, including microRNAs (miRNAs),^18^ long ncRNAs (lncRNAs)^19^ and circular RNAs (circRNAs).^20^ The m6A‐related protein is also affected by ncRNAs.^21^, ^22^ The interplay between m6A and ncRNAs is responsible for the biological processes of many diseases.^23^

Musculoskeletal disorders (MSD) are a class of inflammatory and degenerative diseases caused by motor organ injury or pain, predominantly affecting the musculoskeletal system, such as muscle, bone, cartilage and joint,^24^ and include intervertebral disc degeneration (IDD), osteoporosis (OP), osteosarcoma (OS) and osteoarthritis (OA). Recently, the incidence of MSD continues to rise, and MSD has become the leading cause of disability worldwide, thereby placing a heavy burden on global health and social security system.^25^, ^26^ However, the precise pathogenesis remains unclear, and no effective treatment and drug is available at present.^27^ Accumulating evidence documented that ncRNA m6A modification is closely associated with the musculoskeletal system and is suggested as crucial regulators involved in bone osteogenic^28^ and osteoclastogenic processes^29^ and myogenesis.^30^ The interplay between the m6A‐related protein and ncRNAs play a vital part in the pathological and physiological processes of MSD.^31^, ^32^, ^33^ m6A can affect the generation and biological function of some important ncRNAs, such as pri‐miRNA processing^31^ and maturation^34^ and lncRNA degradation.^35^ NcRNAs (i.e., miRNAs,^36^ lncRNAs and circRNAs^37^) can target or modulate the m6A‐related protein regulating the occurrence and development of MSD.^31^ In this review, we highlight the functional interactions between m6A regulators and ncRNAs in the bone osteogenic and osteoclastogenic processes and myogenesis, further describe the related molecular mechanism of the interplay between m6A and ncRNAs in MSDs, including IDD, OP, OS and OA.

## 2 m6A MODIFICATION

The process of m6A methylation is dynamic and reversible. This process involves three crucial molecular compositions, i.e., m6A methyltransferases (writers), m6A demethylases (erasers) and m6A recognition factors (readers),^38^ which can add, remove and recognize m6A sites, respectively, and are essential for normal biological processes and development in human tissues and cells.^12^ Writers predominantly initiate the m6A modification process, which includes methyltransferase‐like 3/14/16 (METTL3/14/16),^39^, ^40^ zinc finger CCCH‐type containing 13 (ZC3H13), CCHC zinc‐finger‐containing protein ZCCHC4, Wilm's tumour‐associated protein (WTAP), RNA‐binding motif protein 15/15B (RBM15/15B),^23^ vir‐like m6A methyltransferase associated (VIRMA, also known as KIAA1429) and NOL1/NOP2/Sun domain family member 2 (NSUN2). Erasers, which include alkB homologue 5 (ALKBH5) and fat mass and obesity‐associated protein (FTO),^41^ can reverse m6A methylation. Readers consist of YT521‐B homology (YTH) domain‐containing protein family (i.e., YTHDC1/2 and YTHDF1/2/3),^42^ heterogeneous nuclear ribonucleoprotein (HNRNP) family (i.e., HNRNPA2B1, HNRNPC and HNRNPG),^43^, ^44^ eukaryotic translation initiation factor 3 (eIF3), insulin‐like growth factor‐2 mRNA‐binding proteins 1/2/3 (IGF2BP1/2/3),^45^ and leucine‐rich pentatricopeptide repeat‐containing protein (LRPPRC). They can selectively identify m6A modifications.

**FIGURE 1:**
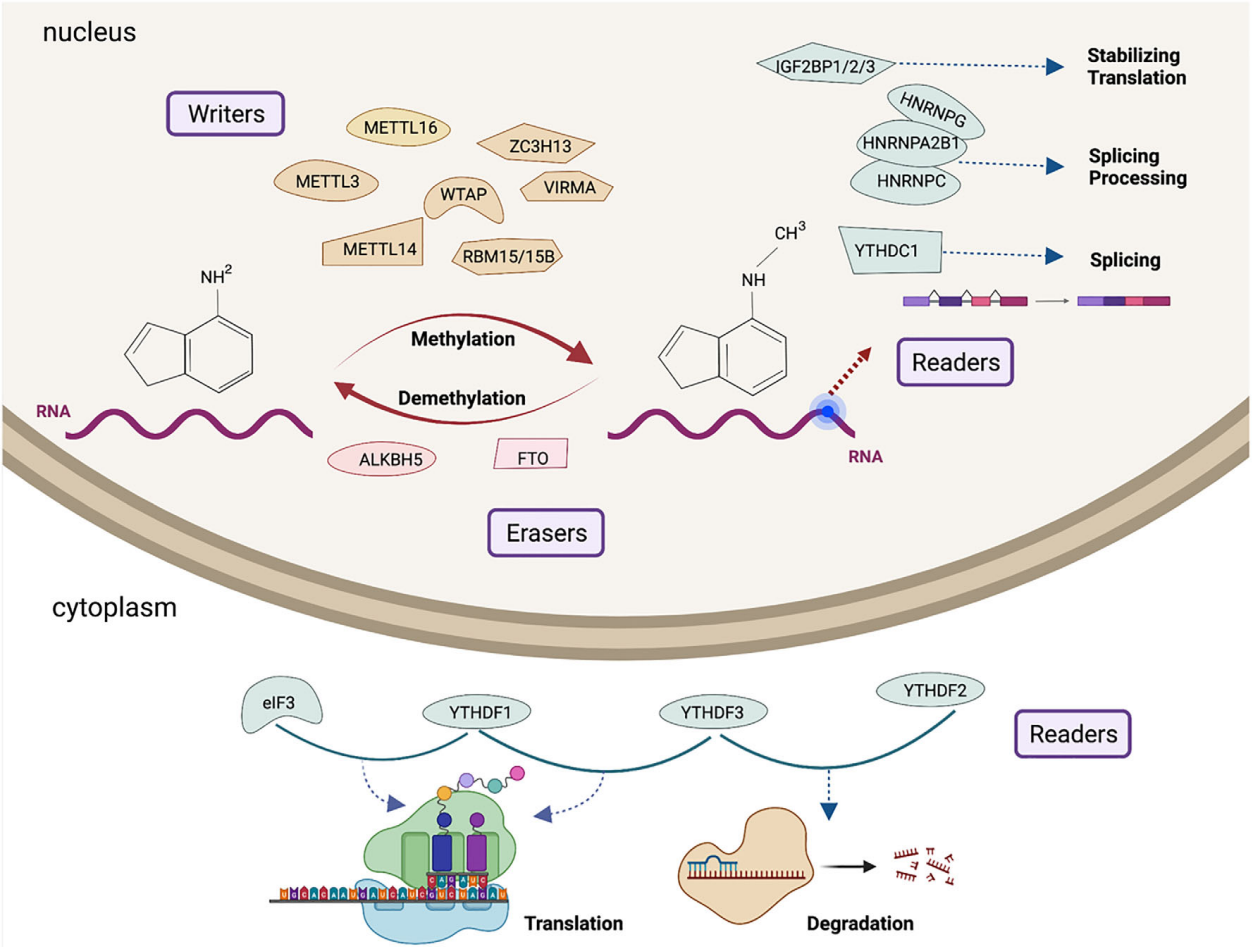
The composition and mechanism of m6A RNA methylation. The process of m6A methylation is dynamic and reversible and involves three molecular compositions: m6A methyltransferases (writers), m6A demethylases (erasers) and m6A recognition factors (readers). m6A RNA modification is highly conserved and is involved in regulating various complex RNA bioprocesse, such as RNA splicing, processing, translation and degradation

m6A RNA modification is highly conserved in humans and mice and is involved in regulating various complex RNA bioprocesses,^46^ such as RNA splicing, processing, translation and degradation.^47^ mRNA precursors (pre‐mRNAs) form mature mRNAs through splicing. METTL16 induce efficient splicing and regulate S‐adenosylmethionine (SAM) homoeostasis,^48^ and FTO and ALKBH5 are responsible for the regulation of exon splicing.^49^ m6A readers (i.e., HNRNPA2B1, HNRNPC and HNRNPG) can recognize and bind to m6A modification sites, thereby regulating RNA alternative splicing.^50^, ^51^ Similar to METTL3, HNRNPA2B1 can interact with RNA‐binding protein DiGeorge syndrome critical region gene 8 (DGCR8) to promote primary miRNA processing.^52^ m6A readers are the main translation and degradation regulator. YTHDF1 can interact with eIF3 to facilitate m6A‐modified mRNA translation and further improve translation efficiency.^53^, ^54^ As the initiator of mRNA translation, YTHDF3 can synergistically promote mRNA translation with YTHDF1.^55^ However, when YTHDF3 interacts with YTHDF2, the decay of m6A mRNA is accelerated.^56^ YTHDF2 can also selectively recognize m6A modification sites and directly induce mRNA degradation.^57^ In addition, another class of m6A readers, i.e., IGF2BP family (including IGF2BP1/2/3), is beneficial to enhance the stability of mRNA and translation^58^ (Figure 1).

Accumulating evidence documented that m6A is closely associated with MSDs and that aberrantly expressed writers,^31^, ^33^, ^59^ erasers^60^, ^61^ and readers^62^ participate in the pathophysiological process of MSD. In IDD, METTL14 is highly expressed in the nucleus pulposus (NP) of patients with IDD. METTL14 could induce NP cell death by modulating interleukin (IL)‐1β and IL‐18 expression levels,^63^ and the overexpressed METTL14 could also promote the senescence of NP cells.^31^ In OP, METTL3, FTO and YTHDF1 mainly regulate osteogenic differentiation of bone marrow‐derived mesenchymal stem cells (BMSCs), thereby promoting OP progression. Yan et al. reported that METTL3 is downregulated in OP; it directly targets RUNX2 and restrains BMSCs osteogenic differentiation.^33^ FTO is overexpressed in bone marrow and promotes BMSCs adipocyte differentiation and restrains osteoblast differentiation by GDF11–FTO–Pparg axis, thereby inhibiting bone formation and accelerating the development of osteopenia.^60^ Wang et al. further reported that FTO could also target RUNX2 directly, thereby inhibiting osteogenic differentiation and promoting the progression of OP.^64^ Liu et al. found that YTHDF1 is upregulated, and it could bind to ZNF839 and the downstream target RUNX2 facilitating BMSCs osteogenesis.^62^ In OS, the most studied m6A‐related proteins are METTL3, WTAP and ALKBH5. They mainly regulate the proliferation, apoptosis, migration and invasion of OS cells, and participate in the progression of OS. METTL3 functions as oncogene and is highly expressed in OS. Studies reported that METTL3 regulated OS cells proliferation, migration and invasion via ATPase family AAA domain‐containing protein 2 (ATAD2)^65^ and LEF1/Wnt/β‐catenin signalling pathway.^66^ Furthermore, METTL3 could also up‐regulate the expression of developmentally regulated GTP‐binding protein 1 (DRG1) promoting OS cells migration and colony formation.^67^ WTAP is highly expressed in osteosarcoma tissue and regulats the proliferation and metastasis of osteosarcoma by PI3K/AKT pathway.^68^ ALKBH5 is abnormally expressed in OS tissues and is crucial for the growth and metastasis of OS. The overexpressed ALKBH5 upregulated the expression of ubiquitin‐specific protease 22 (USP22) and RNF40, and restrained histone H2A ubiquitination, facilitating OS progression.^69^ Furthermore, ALKBH5 could also regulated OS cells proliferation, apoptosis and cycle arrest by targeting selective signal transducer and activator of transcription 3 (STAT3) and suppressor of cytokine signalling 3 (SOCS3).^70^ In OA, the most studied m6A‐related proteins are METTL3, which promotes OA progression by regulating extracellular matrix (ECM) degradation and fibroblast‐like synoviocytes (FLS) senescence. Sang et al. found that METTL3 overexpression regulated TIMPs and MMPs expression affecting the inflammatory response and ECM degradation.^71^ Chen et al. reported that the excessive METTL3 inhibits the autophagy of FLS and promotes the expression of senescence‐associated secretory phenotype, leading to cellular senescence and OA progression.^59^ New study found that FTO are also abnormally expressed in OA. FTO is significantly downregulated in OA cartilage, affecting OA progression by regulating lncRNA AC008.^32^

## 3 ncRNA

NcRNAs are a class of RNAs that have no protein‐coding ability, compose about 98% of mammalian genome^72^ and are previously called transcriptional noise.^73^ In recent years, studies reported that ncRNAs are involved in complex gene transcriptional regulation, such as RNA splicing, processing, editing and translation.^74^ NcRNAs can be divided into two categories in accordance with the regulatory roles. The first category is housekeeping ncRNAs, including ribosomal (rRNAs), transfer (tRNAs), small nuclear (snRNAs), small nucleolar (snoRNAs) and telomerase RNAs, which are necessary for cell viability. The second category is regulatory ncRNAs, which can be further divided into small ncRNAs (< 200 nucleotides nt, include miRNAs, siRNAs and piRNAs) and lncRNAs (> 200 nt). CircRNAs belongs to the two classifications because of their variable length and are involved in regulating transcription and translation processes.^75^ Currently, the most concerned ncRNAs are miRNAs, lncRNAs and circRNAs.

MiRNAs are short ncRNAs with length of 22–23 nt and are predominantly expressed endogenously. MiRNAs can target a third of all human genes, and mRNAs are the main target genes.^76^ In most instances, miRNAs bind to the 3′UTR and act as negative regulators, inhibiting mRNA expression and translation at the post‐transcriptional level and promoting mRNA degradation.^77^ LncRNAs (more than 200 nt) are newly identified ncRNA with a large number^78^ and are key regulators of RNA transcription, splicing, and translation.^77^ CircRNAs are novel endogenous ncRNAs that are approximately 500 nt long and have a stable and conserved unique and closed circular structure.^79^ Thus, circRNAs are considered as ideal biomarkers. Multiple miRNA complementary binding sites are present on circRNAs. circRNAs interact with miRNA participating in transcriptional and post‐transcriptional regulation, which is the main biological function of circRNAs.^80^, ^81^

As key regulators of gene expression, the role of dysregulation ncRNAs in pathophysiological processes of MSDs has been reported gradually. Numerous studies reported that ncRNAs miRNAs, lncRNAs, and circRNAs are aberrantly expressed in IDD,^82^ OP,^83^ OS^84^ and OA.^85^ In IDD, the differentially expressed ncRNAs are involved in regulating cell apoptosis and proliferation, ECM degeneration and inflammation. miR‐338‐3p,^86^ miR‐27a^87^ and miR‐133a‐5p^88^ are upregulated in IDD tissues, and target SIRT6, FSTL1 and FBXO6, respectively, participating in regulating NP cell apoptosis, proliferation and ECM synthesis. The downregulated miR‐181a,^89^ miR‐623^90^ and miR‐874‐3p^91^ are related to inflammatory response and ECM degradation. CircRNAs circRNA_104670,^92^ circ‐4099,^93^ circVMA21,^94^ circ‐GRB10^95^ and circSEMA4B^96^ are involved in cell proliferation and apoptosis of NP. LncRNAs HOTAIR^97^ and LINC00958^98^ are highly expressed and positively correlated with the severity of IDD, act as promoters of NP cell apoptosis and accelerate disease progression. In OP, the differentially expressed ncRNAs regulate osteoblasts and osteoclast differentiation. miRNA‐433‐3p^99^ and miRNA‐139‐5p^100^ and circRNAs CDR1as^101^ and circRNA_0016624^102^ can promote osteoblast differentiation via targeting DKK1, NOTCH1, GDF5 and BMP2, respectively. For osteoclast differentiation, miR‐125a‐5p can promote osteoclast differentiation by inhibiting the expression of the downstream target gene TNFRSF1B.^103^ The lncRNA Bmncr regulates Acp5, Ctr and MMP9 expression levels and inhibits osteoclast differentiation, thus slowing the progression of OP.^104^ CircRNA_28313 induces osteoclast differentiation by circRNA_28313/miR‐195a/CSF1 network.^105^ In OS, the differentially expressed ncRNAs play vital roles in OS cell apoptosis, invasion, growth and migration. The downregulated miR‐193a‐3p targets Rab27B and SRR inhibiting the migration and invasion of OS cells.^106^ The lncRNA TTN‐AS1 is upregulated in OS, promotes cell viability and suppresses apoptosis via the miR‐134‐5p/MBTD1 axis.^107^ The highly expressed circ_0001658 sponges miR‐382‐5p to regulate the expression level of the downstream gene YB‐1, thereby facilitating the proliferation, invasion and migration of OS cells.^108^ In OA, the differentially expressed ncRNAs participate in regulating chondrocyte proliferation, apoptosis, ECM degradation and inflammation. The overexpressed miR‐384‐5p can suppress chondrocyte apoptosis and facilitate chondrocyte proliferation by SOX9 and the NF‐κB signalling pathway.^109^ The lncRNA PVT1 can target miR‐149^110^ and miR‐488‐3P,^111^ thus promoting chondrocyte apoptosis, ECM degradation and inflammatory response. circRNA.33186 and circSERPINE2 are overexpressed in OA, circRNA.33186 binds to miR‐127‐5p and upregulates MMP13 expression, thereby accelerating OA progression.^112^ CircSERPINE2 targets miR‐1271‐5p and plays an important role in regulating proliferation, apoptosis and anabolism of ECM.^113^

## 4 INTERPLAY BETWEEN m6A AND ncRNA

Many m6A modification sites exist in ncRNAs (including miRNAs, lncRNAs, circRNAs, rRNAs, snoRNAs and snRNAs).^13^, ^23^, ^114^, ^115^ m6A can highly modify ncRNAs, which can regulate the expression and function of m6A‐related proteins.^116^ The mutual regulation between m6A and ncRNAs is involved in regulating the biological processes of many diseases.^23^, ^46^

### 4.1 Mutual regulation between miRNA and m6A

Abundant miRNA target sites are found in 3′UTRs where m6A is highly enriched, confirming the strong correlation between m6A and miRNA.^13^ A series of studies indicated that m6A modification regulates miRNA processing, splicing and maturation. DGCR8 is the key participant in the biosynthesis of miRNAs. In the nucleus, DGCR8 breaks down primary miRNA (pri‐miRNA) and converts it into precursor miRNA (pre‐miRNA).^117^ In the cytoplasm, Dicer cleaves pre‐miRNA into single‐stranded miRNAs.^118^ METTL3 acts as an RNA marker that promotes the initiation of miRNA biogenesis by DGCR8 and Dicer. METTL3 accelerates pri‐miRNA processing and maturation via promoting the integration between DGCR8 and pri‐miRNA^119^ and improves miRNA splicing by the pre‐miRNA Dicer cleavage.^120^ Similar to METTL3, METTL14 can interact with DGCR8 and accelerate pri‐miRNA processing.^121^ ALARCÓN and his colleagues identified that the m6A reader HNRNPA2B1 can interact with DGCR8 to activate pre‐miRNA processing. Furthermore, Alarcón et al. found that HNRNPA2B1 can combine with nuclear transcripts and induce alternative splicing as METTL3, but the specific regulatory mechanism remains unclear.^52^ Notably, m6A methylation can negatively regulate miRNA biosynthesis. The knockdown of the m6A eraser FTO reduces the state levels of almost all methylated miRNAs.^122^ However, the expression level of pri‐miRNAs is not changed, and the negative regulation of m6A on miRNA is still unknown and needs further study.

In the classical miRNA regulatory mechanisms, miRNAs interact with downstream target mRNA via complementary base pairing and participate in the negative regulation of mRNA degradation and translation.^123^ Research demonstrated that m6A‐related proteins can act as downstream target genes and can be regulated directly by miRNAs. The 3′UTR of METTL3 mRNA is directly targeted by miR‐33a, thereby restraining non‐small‐cell lung carcinoma cell proliferation.^124^ miR‐145 can target the 3′UTR of YTHDF2 mRNA and regulate m6A levels in hepatocellular carcinoma.^125^ Furthermore, miRNA plays a negative role in inhibiting the translation of m6A‐related protein‐encoding genes. miR‐96 can block m6A modification, increase the expression of m6A eraser FTO and facilitate the c‐myc proto‐oncogene (MYC) expression involved in colorectal cancer (CRC) cell proliferation and apoptosis.^126^

**FIGURE 2:**
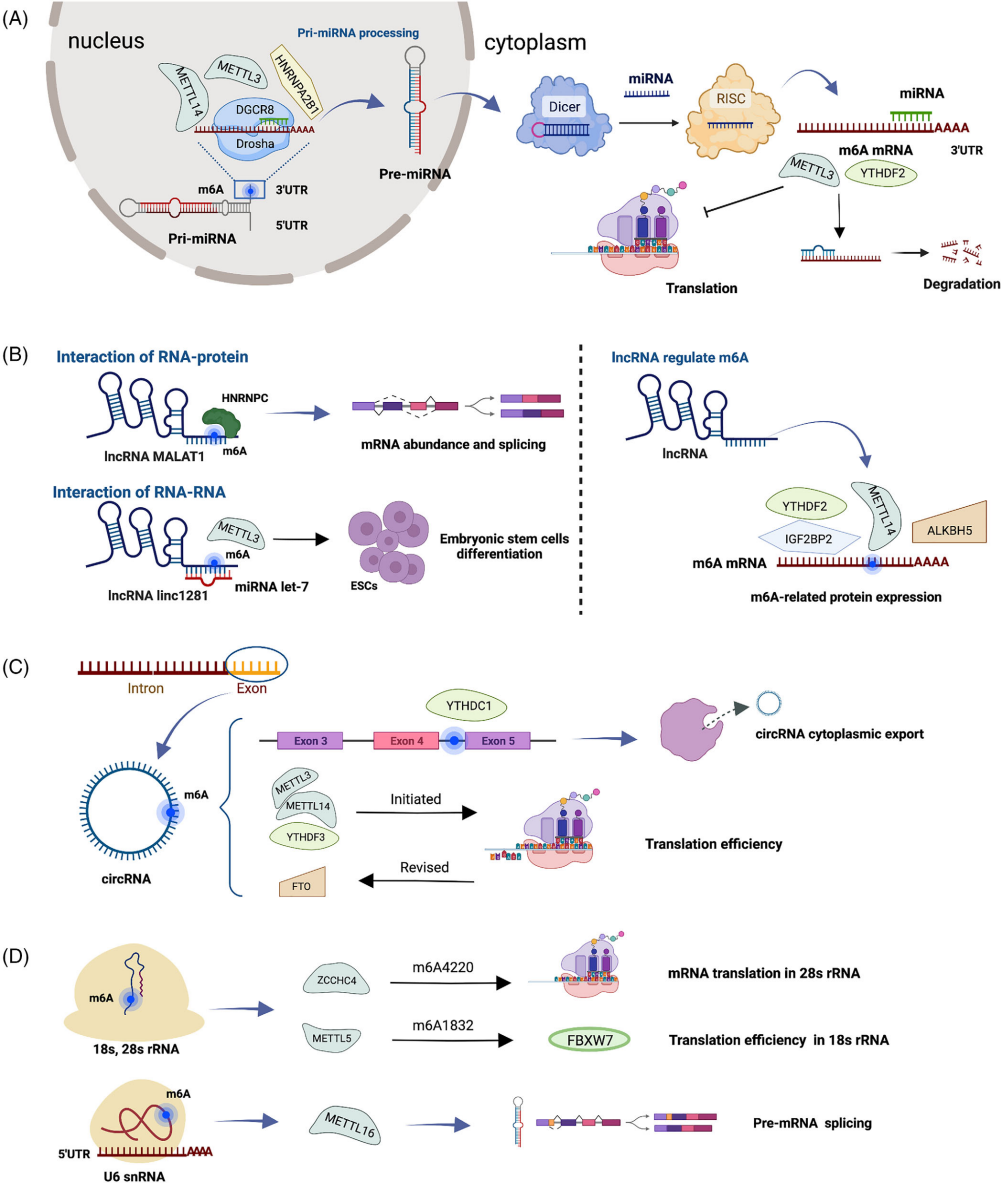
Mutual regulation between m6A and ncRNAs. (A) Mutual regulation between miRNA and m6A. m6A is enriched in miRNA 3′UTRs and regulates miRNA processing, degradation and translation. METTL3, METTL14 and HNRNPA2B1 accelerate pri‐miRNA processing in the nucleus by DGCR8 and drosha. MiRNA targets METTL3 and YTHDF2 that regulate mRNA degradation and translation. (B) Mutual regulation between lncRNA and m6A. m6A modification affects the interactions of RNA–protein and RNA–RNA by the ceRNA network. LncRNA MALAT combines with HNRNPC regulating transcriptome‐wide mRNA abundance and alternative splicing. METTL3 promotes lncRNA linc1281 methylation and mediates linc1281 binding to miRNA let‐7, regulating ESC differentiation. LncRNA can regulate the expression and function of m6A‐related proteins METTL14, YTHDF2 and IGF2BP2. (C) Mutual regulation between circRNA and m6A. m6A‐circRNA modification is modified in exons, YTHDC1 binds to circRNA at the exon 5–exon 4 junction site modulating circRNA cytoplasmic export. METTL3, METTL14 and YTHDF3 initiate circRNA protein translation and reversed by FTO. (D) Mutual regulation between rRNA/snRNA and m6A. ZCCHC4 regulated m6A4220 modification in 28S rRNA involving in mRNA translation, and METTL5 modulated FBXW7 translation efficiency in 18S rRNA A1832. METTL16 is located in U6 snRNA 5′UTR and modulates pre‐mRNAs splicing

In conclusion, the mutual regulation between m6A and miRNA involves two parts. On the one hand, m6A modification accelerates pri‐miRNA processing and maturation via promoting the integration between DGCR8 and pri‐miRNA and improves miRNA splicing by pre‐miRNA Dicer cleavage. On the other hand, miRNA targets the 3′UTR of m6A mRNA and negatively regulates m6A abundance (Figure 2A).

### 4.2 Mutual regulation between lncRNA and m6A

Similar to mRNA, m6A peaks are enriched in lncRNA,^127^ and m6A residues are preferentially localized in lncRNA transcripts.^46^ Recently, the interaction between m6A and lncRNA has been studied extensively. m6A is related to the stability and degradation of lncRNA. Ban and his colleagues reported that m6A METTL3 and METTL14 improve the stability of lncRNA LNCAROD in head and neck squamous cell carcinomas.^128^ Ni et al. found that the m6A reader YTHDF3 selectively binds to lncRNA GAS5 and leads to GAS5 degradation in CRC.^129^ m6A regulates lncRNAs in two ways. (1) m6A modification affects the RNA–protein interaction. LncRNA metastasis‐associated lung adenocarcinoma transcript 1 (MALAT1) is located in the nucleus and binds to various splicing regulators, such as serine/arginine proteins and participates in the alternative splicing of pre‐mRNAs.^130^ New evidence indicated that the m6A located in MALAT1 can increase the accessibility of the U5‐tract via altering a local change in structure of mRNA, thereby facilitating the combination of MALAT with HNRNPC and regulating the transcriptome‐wide mRNA abundance and alternative splicing finally.^131^ This regulatory mechanism is also known as m6A‐switches, which are associated with mRNA splicing, maturation and transcription.^132^ (2) m6A modification affects the RNA–RNA interaction. The vital regulatory mechanism of lncRNA is that lncRNAs bind to miRNAs by the competitive endogenous RNA (ceRNA) network.^133^ Research demonstrated that m6A can regulate the function of lncRNA via the same mechanism and acts as a positive mediator modulating the lncRNA–miRNA interaction by the ceRNA network.^134^ For example, linc1281 is embryonic stem cell (ESC)‐specific RNA and is abundant in mouse ESCs, and m6A modification is highly enriched in the last exon of linc1281.^115^ Yang et al. revealed that the m6A writer METTL3 promotes linc1281 methylation, mediates linc1281 binding to let‐7 miRNAs by the ceRNA network and regulates the expression of the downstream target gene Lin28, thereby regulating ESC differentiation.^115^

The regulatory role of lncRNAs on m6A methylation has been gradually revealed. LncRNAs can modulate the function and expression of m6A‐related proteins and is involved in the pathophysiological process of many diseases. Wang et al. found that the knockdown of lncRNA LINRIS decreases the expression of the m6A reader IGF2BP2 and suppresses the downstream effects of IGF2BP2, thereby regulating the proliferation of CRC.^135^ The m6A eraser ALKBH5 is involved in lncRNA‐mediated m6A modification. The lncRNA GAS5‐AS1 regulates the expression of GAS5 by interaction with ALKBH5. Furthermore, m6A‐mediated GAS5 RNA degradation is closely related to the YTHDF2‐dependent pathway.^136^ The lncRNA LNC942 can recruit the m6A writer METTL14 directly and regulate the expression of downstream targets by post‐transcriptional m6A modification, thereby participating in the initiation and progression of breast cancer.^137^

In conclusion, the mutual regulation between m6A and lncRNA predominantly involves two parts. On the one hand, m6A modification affects the RNA–protein and the RNA–RNA interactions by the ceRNA network. On the other hand, lncRNAs modulate the function and expression of m6A‐related proteins (Figure 2B).

### 4.3 Mutual regulation between circRNA and m6A

In addition to miRNAs and lncRNAs, circRNAs can be modified by m6A.^114^ m6A‐circRNAs methylation has some unique characteristics that are significantly different from mRNA methylation. m6A‐circRNA modification is frequently modified in exons, and circRNAs modified by m6A are highly specific to cell. m6A modification can regulate circRNA cytoplasmic export, translation and degradation.^114^ Chen et al. reported that YTHDC1 can bind to the circRNA circNSUN2 at the exon 5–exon 4 junction site and play roles in modulating circNSUN2 cytoplasmic export in an m6A‐dependent manner.^138^ m6A modification can drive translation initiation and regulate the translation efficiency by m6A level. Yang et al. revealed that m6A promotes circRNA protein translation in a cap‐independent fashion via the internal ribosomal entry site pathway. The translation process is driven by METTL3 and METTL14, reversed by FTO and initiated by YTHDF3.^55^ No functional enrichment is detected even though these circRNAs are enhanced. Increasing studies are needed to explain the translation mechanisms. circRNA is stable and conserved because of its closed circular structure. However, a study found that circRNA can be degraded by the m6A reader YTHDF2 via the endoribonucleolytic cleavage pathway.^139^ The RNase P/MRP (endoribonucleases) is a key enzyme that initiates circRNA cleavage. circRNA interacts with YTHDF2 and is downregulated by the RNase P/MRP in a heat‐responsive protein12 (HRSP12)‐dependent manner. Notably, HRSP12 predominantly acts as adaptor protein in the process of circRNA cleavage. HRSP12 combines YTHDF2 and RNase P/MRP to improve the efficiency of endoribonucleolytic cleavage.^140^

Studies on the regulation of m6A by circRNA are few. Huang et al. reported the functional link of ALKBH5 and circSTAG1 in major depressive disorder (MDD). ALKBH5 and circSTAG1 are downregulated in MDD mouse. Overexpressed circSTAG1 can bind with ALKBH5 and reduce the translocation of ALKBH5 into the nucleus, facilitate fatty acid amide hydrolase methylation and ameliorate depressive‐like behaviours.^141^ In MSD, studies on the regulation of m6A by circRNA are not available and may be the direction for future research (Figure 2C).

### 4.4 Mutual regulation between rRNA/snRNA and m6A

Other ncRNAs, such as rRNA and snRNA, can also be modified by m6A methylation.^142^ Research demonstrated that m6A modification is found on human 28S and 18S rRNAs,^143^, ^144^ but the enzyme responsible for rRNA m6A methylation is still unclear. A recent study has found that ZCCHC4 is identified as the m6A4220 enzyme involved in the deposition of human 28S rRNA and regulation of mRNA translation.^145^ ZCCHC4 is localized in nucleolus; ZCCHC4 knockout inhibited m6A4220 modification in 28S rRNA and reduced mRNA translation.^145^, ^146^ METTL5 is the m6A methyltransferase responsible for the 18S rRNA. METTL5 methylated 18S rRNA A1832 and regulated the translation efficiency of F‐box and WD repeat domain‐containing 7 (FBXW7).^147^ Different from conventional m6A RNA methyltransferase, METTL5 must bind to TRMT112 to form a stable heterodimeric complex, thereby forming m6A1832 in 18S rRNA.^148^ Post‐transcriptional modifications on snRNA are also demonstrated. Studies confirmed that m6A modification is on position 43 located in the evolutionarily conserved U6 sequence^48^ and A43 base pairs with 5′ splice sites of pre‐mRNAs during splicing.^149^ METTL16 is the methyltransferase for the U6 spliceosomal snRNA participating in the regulation of splicing.^149^ Another study found that METTL16 regulates methionine adenosyltransferase 2A expression in humans by enhancing intron‐preserving splicing when SAM is lacking^48^ (Figure 2D). Overall, although m6A methylation has been found in a variety of ncRNAs, the biological functions and regulatory roles in diseases are still poorly understood. More research is needed in the future.

## 5 INTERPLAY BETWEEN m6A AND ncRNA IN MUSCULOSKELETAL BIOLOGY

The musculoskeletal system includes bones and skeletal muscles. Bone loss and sarcopenia are the main clinical manifestations of MSD.^150^ In recent years, the role of ncRNA m6A modification in musculoskeletal system has been gradually discovered.

### 5.1 Mutual regulation between m6A and ncRNA in bone

Bone is an important motor organ of human body and is the basis of human movement. Bone is characterized by continuous regeneration and remodelling throughout life to maintain and repair bone during homoeostasis and injury.^151^ The process of bone regeneration and remodelling involves two stages, i.e., bone formation and resorption.^152^ Bone formation is predominantly mediated by osteoblasts, which are key factors in matrix synthesis and bone mineralization. Bone resorption is mediated by osteoclasts whose primary function is the removal of bone mass.^153^ Bone formation and resorption are in a dynamic balance, and osteoblasts interact with osteoclasts to maintain stable bone mass.^152^ When the bone homoeostasis is upset, it can lead to a range of diseases, such as RA,^154^ OP^155^ and OA.^156^ The maintenance of bone homoeostasis depends on the differentiation potential of BMSCs.^157^ BMSCs are pluripotent nonhaematopoietic stem cell population that can differentiate into chondrocytes, adipocytes, osteocytes, osteoblasts and myoblasts, ultimately generating bone, cartilage and fat tissues.^158^ Therefore, the role of BMSCs in bone and cartilage development and repair has gained attention.

New evidence indicated that ncRNA m6A modification is closely associated with BMSC osteogenic differentiation.^159^, ^160^ The mechanism is that miRNA directly targets m6A demethylase FTO and regulates BMSC osteogenic differentiation. Li et al. found that miR‐149‐3p is overexpressed in the osteoblastic differentiation period, and FTO is the direct target of miR‐149‐3p. Upregulated miR‐149‐3p significantly downregulates FTO mRNA expression and promotes the osteogenic differentiation of BMSCs.^159^ Zhang and his colleagues reported that miR‐22‐3p is a positive regulator of osteogenic differentiation and can increase RUNX2, osteocalcin and osteopontin expression levels during BMSC osteogenic differentiation. Furthermore, upregulated miR‐22‐3p targets FTO and negatively regulates FTO methylation via the MYC/PI3K/AKT pathway, accelerating osteogenic differentiation.^160^ Similar to FTO, METTL3 can promote osteogenic differentiation by regulating ncRNA. Song et al. revealed that METTL3 is upregulated in human adipose‐derived stem cells (hASCs). As osteogenesis promotor, METTL3 targets lncRNA RP11‐44 N12.5 and regulates the RP11‐44 N12.5 m6A modification by activating the STK3/MAPK signalling pathway, thereby promoting hASC osteogenic differentiation.^28^ However, another study found that METTL3 acts as an inhibitor of osteogenic processes. METTL3 inhibits miR‐7212‐5p maturation by the METTL3/miR‐7212‐5p/FGFR3 axis, restraining osteoblast differentiation.^161^ Notably, a recent study has found that METTL3 can regulate lncRNA–miRNA–mRNA axis to promote osteogenic differentiation. Yuan et al. found that lncRNA XIST negatively regulates miR‐302a‐3p expression and that ubiquitin‐specific peptidase 8 (USP8) is the target gene of miR‐302a‐3p. METTL3 promotes the ossification of primary ligament fibroblasts by the XIST/miR‐302a3p/USP8 axis.^162^ The main mechanism of lncRNAs is that lncRNAs act as miRNAs sponge competitively binding to miRNAs and inhabiting miRNA expression.^163^ However, in this study, whether lncRNA acts as miRNA sponge is not clear.

**FIGURE 3:**
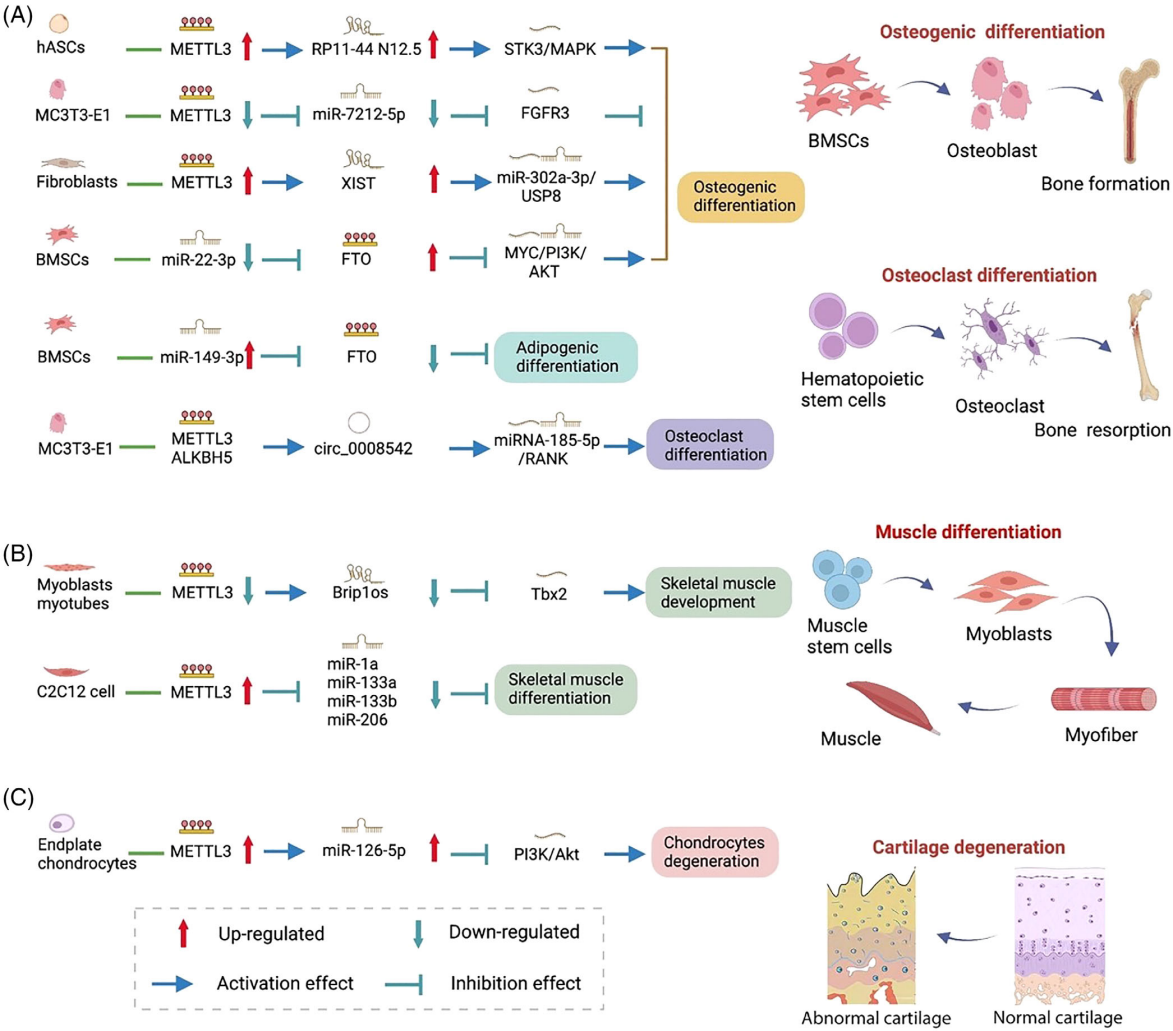
Interplay between m6A and ncRNA in bone and skeletal muscle. ncRNA m6A methylation in bone (A), skeletal muscle (B) and cartilage (C)

The m6A‐circRNA methylation is also associated with osteoclast differentiation and bone resorption. Wang et al. revealed multiple potential m6A sites in circ_0008542–9, and METTL3 and ALKBH5 can regulate circ_0008542–9 m6A methylation levels. In RAW264.7 cells, the exosome circ_0008542 competitively binds to miRNA‐185‐5p acting as miRNA sponge and enhances the expression of the target Tnfrsf11a (RANK), thereby promoting osteoclast differentiation and bone resorption. The m6A methylase METTL3 can promote the competitive binding of circ_0008542 to miRNA‐185‐5p and lead to the initiation of osteoclast bone absorption by the circ_0008542/miRNA‐185‐5p/RANK axis. The process that METTL3 regulates the binding efficiency between circRNA and miRNA and changes the spatial structure of circ_0008542 by circRNA m6A functional site is called “m6A‐switch”.^29^ The “m6A‐switch” is the regulation mechanism of RNA–protein interactions by m6A‐dependent RNA structural remodelling.^132^ Currently, studies on m6A ncRNA methylation related to “m6A‐switch” are few and may be the direction for future research (Figure 3A and Table 1).

**TABLE 1:** Interplay between m6A and ncRNA in bone and skeletal muscle

m6A component	Expression	ncRNA	Expression	Interplay	Pathway	Function	References
Bone/osteogenic differentiation							
METTL3	Up	lncRNA RP11‐44 N12.5	Up	METTL3 regulate RP11‐44 N12.5	METTL3/RP11‐44 N12.5/STK3/MAPK	Enhancing osteogenic differentiation of hASCs	[28]
METTL3	Down	miRNA miR‐7212‐5p	Down	METTL3 target miR‐7212‐5p	METTL3/miR‐7212‐5p/FGFR3	Inhibiting osteogenic differentiation	[161]
METTL3	Up	lncRNA XIST	Up	METTL3 regulate lncRNA XIST	XIST/miR‐302a‐3p/ USP8	Enhancing osteogenic differentiation of fibroblasts	[162]
FTO	Down	miRNA miR‐149‐3p	Up	miR‐149‐3p target FTO	miR‐149‐3p/FTO	Inhibiting the adipogenic differentiation of BMSCs	[159]
FTO	Up	miRNA miR‐22‐3p	Down	miR‐22‐3p target FTO	miR‐22‐3p/FTO/ MYC/PI3K/AKT	Promoting osteogenic differentiation	[160]
Bone/osteoclast differentiation							
METTL3 ALKBH5		circRNA circ_0008542	–	METTL3/ALKBH5 regulate circ_0008542	METTL3/circ_0008542/miRNA‐185‐5p/ RANK	Initiating osteoclast bone absorption	[29]
Cartilage							
METTL3	Up	miRNA miR‐126‐5p	Up	METTL3 regulate miR‐126‐5p	METTL3/miR‐126‐5p/PI3K/Akt	Accelerating the degeneration of endplate chondrocytes	[164]
Skeletal muscle							
METTL3	Up	miRNAs miR‐1a/miR‐133a/miR‐133b/miR‐206	Down	METTL3 regulate miRNAs	METTL3/miRNAs	Repressing the muscle‐ specific miRNAs expression	[30]
METTL3	Down	lncRNA Brip1os	Down	METTL3 regulate Brip1os	METTL3/Brip1os/ Tbx2	Promoting skeletal muscle development	[171]

In addition to bone formation and resorption, the m6A modifications of ncRNA is involved in regulating cartilage function. Xiao et al. found that METTL3 and miR‐126‐5p are upregulated in endplate chondrocytes induced by IL‐1β and that METTL3 regulates the expression of miR‐126‐5p and downstream target phosphatidylinositol‐3‐kinase regulatory subunit 2 (PIK3R2) by the PI3K/Akt pathway, leading to dysfunctional cell vitality and metabolism and accelerating the degeneration of endplate chondrocytes^164^ (Figure 3C and Table 1).

### 5.2 Mutual regulation between m6A and ncRNA in skeletal muscle

The skeletal muscle is an important functional organ in the human body, accounting for about 30%–40% of the body weight, and plays a key role in human metabolism, endocrine system and voluntary movement.^165^ Skeletal muscle is composed of multinucleated muscle fibres, and mononucleated myoblasts fuse together in longitudinal arrays to form mature muscle fibres.^166^ The skeletal muscle has regenerative capacity. When the muscle is injured, muscle satellite cells, which are muscle stem cells, are activated, thus multiplying and differentiating into myoblasts. These myoblasts move to the injured area and fuse with damaged muscle fibres to promote muscle formation, growth and repair.^166^, ^167^ However, the dysregulation of proliferation and differentiation of muscle cells during myogenesis lead to impaired skeletal muscle function, causing a range of MSDs.^168^ The in‐depth exploration of skeletal muscle functional mechanism is important for understanding the pathophysiological basis and diagnosis and treatment of MSD.

The functional regulation of ncRNA and m6A methylation in skeletal muscle development, differentiation and maintenance of muscle homoeostasis has been reported widely.^169^, ^170^ New evidence indicated that muscle‐specific ncRNAs are regulated by m6A RNA modification at the post‐transcriptional level.^30^, ^171^ Diao et al. found that pri‐miRNA sequences are significantly enriched in conserved m6A modification motifs. The m6A writer METTL3 is overexpressed in muscle injury and regeneration mouse model, whereas muscle‐specific miRNAs miR‐1a, miR‐133a, miR‐133b and miR‐206 are downregulated. METTL3 can inhibit the expression levels of these miRNAs by the m6A modification of pri‐miRNAs. Furthermore, METTL3 acts as negative regulator in myoblast state transition and represses muscle‐specific miRNA expression, thereby reducing the differentiation of C2C12 myoblasts.^30^ In addition to miRNA, lncRNA is regulated by METTL3 in the differentiation of C2C12 myoblasts. Xie et al. detected abundant differentially expressed lncRNAs and m6A methyltransferases and demethylases during C2C12 differentiation. m6A motifs are significantly enriched in lncRNA, and m6A methylation levels are positively correlated with m6A‐modified lncRNAs. In the process of muscle tissue development, m6A methylates lncRNAs and regulates the expression of nearby mRNAs. The lncRNA Brip1os and m6A methyltransferase METTL3 are downregulated, whereas Brip1os near the mRNA Tbx2 is upregulated. METTL3 regulates skeletal muscle development by the METTL3/Brip1os/Tbx2 axis.^171^ These studies showed that METTL3 regulates muscle‐specific ncRNAs via the post‐transcriptional level, playing an important role in skeletal muscle differentiation and development. METTL3 can also modulate muscle‐specific miRNAs via the transcriptional level. A study found that METTL3 directly regulates muscle cell differentiation‐related transcription factors (i.e., MEF2A, MEF2C and SRF) and epigenetic regulators (i.e., HDAC1, HDAC4 and HDAC8) repressing the expression level of miRNAs.^30^ Overall, these studies revealed crosstalk between m6A and ncRNA during skeletal muscle differentiation and development, providing new insights into ncRNA m6A modification in skeletal muscle and MSD (Figure 3B and Table 1).

## 6 INTERPLAY BETWEEN m6A AND ncRNA IN MSDs

MSDs affect muscle, bone, cartilage and joint, causing human body's movement and musculoskeletal dysfunctions.^172^ However, cellular and molecular mechanisms remain unclear. The mutual regulation between m6A and ncRNA is found in MSD, and m6A modification can regulate ncRNAs, which ncRNAs can also target m6A regulators to influence MSD pathological and physiological processes.

### 6.1 Mutual regulation between m6A and ncRNA in IDD

**FIGURE 4:**
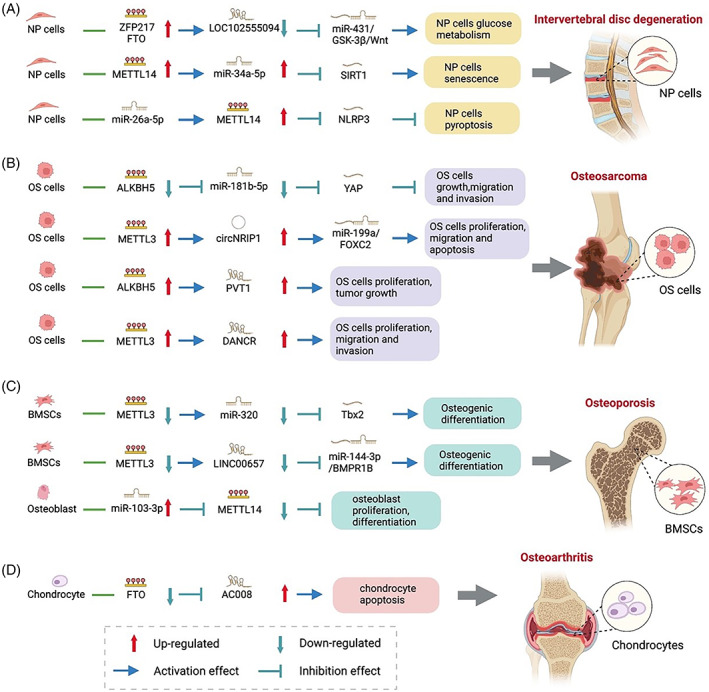
The interplay between m6A and ncRNA in MSD. ncRNA m6A methylation in IDD (A), OS (B), OP (C) and OA (D)

IDD is a common MSD that is identified to be the main cause of lower back pain and affects 18.3%–30.8% of the global population.^173^, ^174^ The intervertebral disc consists of NP and annulus fibrosus and is located between the upper and lower vertebrae. As a major component of intervertebral disc, abnormal functions of NP cell are the main pathogenesis of IDD.^175^ New evidence indicated that ncRNA m6A modification plays vital roles in regulating the function of NP cell.^31^, ^63^, ^176^ The regulatory mechanism between m6A and lncRNAs is that m6A acts as a positive mediator modulating the lncRNA–miRNA interaction by the ceRNA network. Wang et al. found 261 lncRNAs with significantly differential m6A methylation levels in NP of IDD by epitranscriptomic microarray analysis. The RNA methylation regulator zinc‐finger protein 217 (ZFP217) and m6A demethylase FTO are upregulated in IDD, and ZFP217 can activate FTO transcription and accelerate lncRNA m6A demethylation. The demethylated lncRNA LOC102555094 regulates GSK‐3β expression by binding to miR‐431, regulating glucose metabolism of NP cells and accelerating disc degeneration.^176^ Furthermore, the mutual regulation between m6A and miRNA is found in NP cells, and m6A can regulate the m6A modification of miRNA. The m6A modification accelerates pri‐miRNA processing via promoting the integration between DGCR8 and pri‐miRNA. Zhu et al. reported that METTL14 methylation and miR‐34a‐5p expression are increased in the NP tissues of patients with IDD. METTL14 facilitates the pri‐miR‐34a processing via regulating the recognition and binding of DGCR8 and pri‐miR‐34a. SIRT1 is the downstream target of miR‐34a‐5p, and METTL14 promotes miR‐34a‐5p methylation by targeting SIRT1 facilitating NP cell senescence.^31^ Notably, miRNA can target the 3′UTR of m6A mRNA and negatively regulates m6A abundance in NP cells. Yuan et al. found that the overexpressed METTL14 is inhibited by human umbilical cord mesenchymal stem cell (hucMSC) exosomes. NLRP3 is the downstream target of METTL14, and hucMSC‐secreted exosomal miR‐26a‐5p directly degrades METTL14 inhibiting NP cell pyroptosis via the METTL14/NLRP3 inflammatory pathway.^63^ Overall, these studies showed the evidence of the interaction between ncRNA (lncRNA and miRNA) and m6A in the IDD pathophysiological process, providing a new direction for further study (Figure 4A and Table 2).

**TABLE 2:** The interplay between m6A and ncRNA in MSD

m6A component	Expression	ncRNA	Expression	Interplay	Pathway	Function	References
IDD							
ZFP217 FTO	Up	lncRNA LOC102555094	Down	ZFP217 regulate LOC102555094	ZFP217/LOC102555094/miR‐431/GSK‐3β/Wnt	Regulating glucose metabolism of NP cells	[176]
METTL14	Up	miRNA miR‐34a‐5p	Up	METTL14 target miR‐34a‐5p	METTL14/ miR‐34a‐5p/SIRT1	Facilitating senescence of NP cells	[31]
METTL14	Up	miRNA miR‐26a‐5p	–	miR‐26a‐5p target METTL14	miR‐26a‐5p/ METTL14/NLRP3	Inhibiting pyroptosis of NP cells	[63]
OS							
ALKBH5	Down	miRNA miR‐181b‐5p	Down	ALKBH5 target miR‐181b‐5p	ALKBH5/miR‐181b‐5p/YAP	Inhibiting growth, migration and invasion of OS cells	[34]
METTL3	Up	circRNA circNRIP1	Up	METTL3 regulate circNRIP1	METTL3/circNRIP1/ miR‐199a/FOXC2	Promoting proliferation, migration and apoptosis of OS cells	[37]
ALKBH5	Up	lncRNA PVT1	Up	ALKBH5 target PVT1	ALKBH5/PVT1	Promoting OS cells proliferation and tumour growth	[35]
METTL3	Up	lncRNA DANCR	Up	METTL3 regulate DANCR	METTL3/DANCR	Promoting proliferation, migration and invasion of OS cells	[181]
OP							
METTL3	Down	miRNA miR‐320	Down	METTL3 regulate miR‐320	METTL3/miR‐320/ RUNX2	Facilitating BMSCs osteogenic differentiation and bone formation	[33]
METTL3	Down	lncRNA LINC00657	Down	METTL3 regulate LINC00657	METTL3/ LINC00657/ miR‐144‐3p/BMPR1B	Facilitating BMSCs osteogenic differentiation	[187]
METTL14	Down	miRNA miR‐103‐3p	Up	miR‐103‐3p target METTL14	miR‐103‐3p/METTL14	Restraining osteoblast proliferation, differentiation and matrix mineralization	[36]
OA							
FTO	Down	lncRNA AC008	Up	FTO regulate AC008	FTO/AC008/miR‐328‐3p‐AQP1/ANKH	Accelerate chondrocyte apoptosis and ECM degradation	[32]

### 6.2 Mutual regulation between m6A and ncRNA in OS

OS is a common primary malignant bone tumour that predominantly affects long bones. OS occurs mostly in children and adolescents and ranks fifth among those aged 15 to 19 years.^177^ OS is characterized by the abnormal differentiation of mesenchymal stem cells, forming immature bone and osteoid by tumour cells and erosion of bony cortex.^178^ OS attacks the musculoskeletal system, causing joint pain, swelling, muscle atrophy, joint dysfunction and even pathological fracture.^179^ OS has a high degree of heterogeneity and significant genome complexity, and the pathophysiology remains unclear.^180^ The mutual regulation between m6A and miRNA/circRNA/lncRNA is found in OS and is involved in the growth, migration, invasion of tumour cells, tumorigenesis and tumour progression.^34^, ^37^, ^181^ The major mutual regulation mechanism between miRNA and m6A in OA is that m6A accelerates pri‐miRNA processing and maturation. Yuan et al. reported that the m6A demethylase ALKBH5 is downregulated, and m6A methylation is substantially increased in OS cells/tissues. Pre‐miR‐181b‐1 is a potential target of ALKBH5. ALKBH5 methylates pre‐miR‐181b‐1 and accelerates pre‐miRNA maturation in the cytosol. ALKBH5 acts as an antitumour factor that inhibits osteosarcoma cell growth, migration and invasion by dual mechanisms. On the one hand, ALKBH5 directly targets the Yes‐associated protein (YAP) m6A methylation, inhibiting its mRNA stability and translation. On the other hand, ALKBH5 upregulates miR‐181b‐5p and inhibits the downstream target YAP involved in the development and progression of OS.^34^ The mutual regulation between circRNA and m6A is also found in OS, and circRNA m6A modification regulates the development of OA by the circRNA–miRNA–mRNA network. Meng et al. detected that METTL3 and circNRIP1 are upregulated in OS cells/tissues, and circNRIP1 can promote forkhead box protein C2 (FOXC2) expression by sponging miRNA miR‐199a. METTL3 contributes to circNRIP1 m6A modification, promoting cell proliferation and apoptosis by the circNRIP1/miR‐199a/FOXC2 axis.^37^ The regulatory mechanism between m6A and lncRNAs in OS is that m6A modification regulates the stability and degradation of lncRNA. LncRNA PVT1, DANCR, m6A demethylase ALKBH5 and methyltransferase METTL3 are upregulated in OS, and lncRNA PVT1 can associate with ALKBH5, suppressing PVT1 degradation and facilitating PVT1 stability. ALKBH5 promotes OS cell proliferation and tumour growth partially by the ALKBH5–PVT1 axis.^35^ METTL3 can increase lncRNA DANCR mRNA stability by m6A modification, contributing to OS cell proliferation, migration and invasion.^181^ Other m6A‐related lncRNAs are associated with OS. Zhang et al. analysed OS gene expression profiles and identified 111 m6A‐related lncRNAs, which are closely associated with the prognosis of OS.^182^ In summary, the ncRNA m6A modification plays vital roles in OS pathophysiology, and the in‐depth exploration of new m6A‐related ncRNAs in OS may be a promising direction (Figure 4B and Table 2).

### 6.3 Mutual regulation between m6A and ncRNA in OP

OP is a chronic metabolic bone disease that is characterized by bone loss and impaired bone tissue microstructure and bone strength, resulting in increased bone fragility and fracture. OP has become a major global health problem.^183^ The imbalance of bone homoeostasis is the pathogenesis of OP, which is manifested as increased osteoclast bone resorption and decreased osteoblast bone formation.^184^ Promoting BMSC osteogenic differentiation and remodelling bone homoeostasis is the main treatment methods for OP.^185^ Growing evidence showed that ncRNA m6A modification plays vital roles in regulating BMSC osteogenic differentiation; ncRNA and m6A are potential target for OP.^33^, ^36^, ^186^ In BMSCs, METTL3 targets the miRNA that regulates osteogenic differentiation. Yan and his colleagues found that METTL3 is a pro‐osteogenic factor and is downregulated in the bone tissue of patients with OP. METTL3 can methylate pre‐miR‐320 in the nucleus and regulate pre‐miR‐320 maturation in the cytosol. METTL3 downregulates miR‐320 and upregulates the downstream target gene RUNX2, inhibiting BMSC osteogenic differentiation and bone formation.^33^ MiRNA can directly target m6A methyltransferase to inhibit osteoblast activity functionally. Sun et al. reported that miR‐103‐3p acts as negative regulator restraining osteoblast proliferation and matrix mineralization. miR‐103‐3p is upregulated in the bone tissue of OP and negatively regulates METTL14 m6A methylation, thereby inhibiting osteoblast activity. In addition, METTL14 can regulate pri‐miR‐103‐3p processing via promoting DGCR8 and pri‐miR‐103‐3p recognition.^36^ The mutual regulation between m6A and lncRNA is also found in OP. m6A regulates the function of lncRNA via the ceRNA network participating in BMSC osteogenic differentiation. METTL3 is downregulated in OP. LncRNA LINC00657 can compete to bind to miR‐144‐3p and target bone morphogenetic protein receptor B 1 (BMPRB1). METTL3 facilitates BMSC osteogenic differentiation via the LINC00657/miR‐144‐3p/BMPR1B axis in OP.^187^ In addition to miRNA and lncRNA, other ncRNAs, such as piRNA, can be m6A methylated in OP. piRNA m6A modification is involved in osteoblast differentiation. piR‐36741 binds to METTL3 and impedes METTL3‐mediated BMP2 m6A methylation that upregulates the BMP2 expression, thereby promoting BMSC osteogenic differentiation and inhibiting OP progression.^186^ Overall, existing studies illustrated that the mutual regulation between METTL3/METTL14 and miRNA/lncRNA/piRNA plays critical roles in OP by regulating BMSC osteogenic differentiation, which may be potential target for OP (Figure 4C and Table 2).

### 6.4 Mutual regulation between m6A and ncRNA in OA

OA is the most common degenerative joint disease affecting 10%–18% of people over 60 years of age and is a major source of pain and disability worldwide.^188^ The main pathological manifestations are articular cartilage degeneration, subchondral ossification, synovial inflammation and systemic inflammation.^189^ OA is a complex disease that affects the entire joint, and its exact pathogenesis is still unclear. The role of ncRNAs in the pathogenesis of OA has been extensively studied. ncRNA is differentially expressed in articular cartilage and plays vital roles in chondrocyte proliferation, apoptosis, ECM degradation and inflammation.^190^, ^191^ New evidence demonstrated that ncRNA m6A modification is also associated with the pathogenesis of OA, and m6A can regulate RNA stability and ncRNA expression.^32^ Yang et al. detected that lncRNA AC008 is a key regulator of OA, and AC008 can accelerate chondrocyte apoptosis and ECM degradation and inhibit chondrocyte viability by the miR‐328‐3p‐AQP1/ANKH axis. The m6A demethylase FTO is found to be highly enriched in AC008 sequence from chondrocytes, and the overexpressed FTO decreases the m6A level of AC008 and its RNA stability. By contrast, the low FTO improves AC008 RNA stability and upregulates AC008 expression in OA.^32^ This work is the first to study the interaction between ncRNA and m6A in OA, and more studies are needed to discover more ncRNA m6A modification of OA (Figure 4D and Table 2).

## 7 CONCLUSION AND PERSPECTIVES

m6A is the most common internal modification of mRNAs in eukaryotic species, and ncRNA is the major component of the human genome and participates in the regulation of multiple biological processes. The roles of m6A methylation and ncRNA in human diseases have been extensively studied.^192^, ^193^ NcRNA and m6A methylation participate in the pathophysiological process of various human diseases and regulate the occurrence and development of diseases.^194^, ^195^ Recently, with the rapid development of bioinformatics analysis and gene sequencing technology, an increasing number of m6A modification sites are identified in ncRNAs, and ncRNA m6A modification has become a research hotspot and attracted wide attention.

m6A interacts with numerous ncRNAs, modulates multiple biological processes and plays important roles in MSDs, such as IDD, OP, OS and OA. The m6A‐related protein (writers/erasers/readers) can regulate ncRNAs participating in RNA processing, splicing, translation, maturation, RNA–protein interactions and RNA–RNA interactions. Moreover, m6A modifications are regulated by numerous ncRNAs (miRNAs/lncRNAs/circRNAs). NcRNAs regulate the interactions between m6A‐related protein and downstream target mRNA nascent transcripts and control target mRNA degradation, translation and expression.^77^ Studies confirmed that some m6A‐related proteins in ncRNA are abnormally expressed and participate in the occurrence and development of musculoskeletal diseases by modulating the homoeostasis of skeletal muscle, bone and cartilage.^32^, ^187^ However, the regulatory mechanism of ncRNA m6A modification is still unclear. Furthermore, studies mostly focused on the modification of miRNAs and lncRNAs by m6A writers (METTL3/METTL14) and erasers (ALKBH5/FTO), and studies on circRNAs and other m6A‐related protein are few. Thus, further research is needed.

Musculoskeletal diseases are a class of inflammatory and degenerative diseases affecting muscle, bone, cartilage and joint and are the leading cause of disability worldwide. However, effective treatments and drugs are not available at present.^25^ Numerous m6A‐modified ncRNAs, which have been found abnormally expressed in musculoskeletal diseases and participate in the pathophysiological process of diseases, may be promising potential therapeutic targets for diseases. In IDD, hucMSCs deliver exogenous miR‐26a‐5p, which can directly target METTL14 that improves the viability of NP cells and inhibits IDD progression by METTL14/NLRP3 pathways.^63^ Exosomes are small extracellular vesicles that can transfer cargos to reprogramme recipient cells primarily by miRNAs.^196^ Exosomes have been extensively studied in the treatment of tumours through a m6A‐dependent manner,^197^ but studies on musculoskeletal diseases are few. This study provides theoretical support for the application of exosomes in the clinical treatment of musculoskeletal diseases, and exosomes targeting m6A‐related proteins may be a promising therapeutic direction for musculoskeletal diseases.

Overall, studies on the mutual regulation between m6A and ncRNA provide a new perspective for studying the pathophysiological mechanism of musculoskeletal diseases. With more in‐depth studies, more ncRNA m6A molecular regulatory mechanisms will be uncovered, and targeting m6A‐modified ncRNAs may be a promising therapy approach.

## AUTHOR CONTRIBUTIONS

X.Q.W. contributed to conceptualization, supervision and writing—review and editing. X.A.Z. contributed to project administration, funding acquisition and writing—review and editing. J.J.H. contributed to conceptualization, writing—original draft preparation, and visualization. H.K. contributed to writing—original draft preparation. All authors have read and agreed to the published version of the manuscript.

## CONFLICT OF INTEREST

The authors declare no conflict of interest.

## INSTITUTIONAL REVIEW BOARD STATEMENT

Not applicable.

## INFORMED CONSENT STATEMENT

Not applicable.

## Data Availability

Not applicable.
